# Bayesian Network as a Decision Tool for Predicting ALS Disease

**DOI:** 10.3390/brainsci11020150

**Published:** 2021-01-23

**Authors:** Hasan Aykut Karaboga, Aslihan Gunel, Senay Vural Korkut, Ibrahim Demir, Resit Celik

**Affiliations:** 1 Department of Statistics, Amasya University, Amasya 05100, Turkey; 2 Department of Statistics, Yildiz Technical University, Istanbul 34220, Turkey; idemir@yildiz.edu.tr (I.D.); rcelik@yildiz.edu.tr (R.C.); 3 Department of Chemistry, Ahi Evran University, Kirsehir 40200, Turkey; gunel.aslihan@gmail.com; 4 Department of Molecular Biology and Genetics, Yildiz Technical University, Istanbul 34220, Turkey; skorkut@yildiz.edu.tr

**Keywords:** motor neuron disease, amyotrophic lateral sclerosis, Parkinson’s disease, machine learning, Bayesian networks, predictive model

## Abstract

Clinical diagnosis of amyotrophic lateral sclerosis (ALS) is difficult in the early period. But blood tests are less time consuming and low cost methods compared to other methods for the diagnosis. The ALS researchers have been used machine learning methods to predict the genetic architecture of disease. In this study we take advantages of Bayesian networks and machine learning methods to predict the ALS patients with blood plasma protein level and independent personal features. According to the comparison results, Bayesian Networks produced best results with accuracy (0.887), area under the curve (AUC) (0.970) and other comparison metrics. We confirmed that sex and age are effective variables on the ALS. In addition, we found that the probability of onset involvement in the ALS patients is very high. Also, a person’s other chronic or neurological diseases are associated with the ALS disease. Finally, we confirmed that the Parkin level may also have an effect on the ALS disease. While this protein is at very low levels in Parkinson’s patients, it is higher in the ALS patients than all control groups.

## 1. Introduction

Amyotrophic lateral sclerosis (ALS) is a rare neurological disorder mainly caused by progressive degeneration of upper and lower motor neurons. Currently, it is not possible to cure or stop the progression of this disease [1]. ALS may initially affect only one hand or only one leg, making it difficult to walk in a straight line. As the disease progresses, severe muscle weakness, decrease in muscle mass, impaired speech, swallow, fine and gross motor function, and respiratory weakness occur in patients. These lead to paralysis and death usually within 2–5 years following diagnosis [2].

ALS is a multifactorial disease. Approximately 10% of ALS cases are familial (fALS) and 90% of cases are sporadic (sALS) [3]. Although its etiology largely unknown, mutations in various genes have been associated to the ALS [4,5]. There are also some underlying biochemical mechanisms have been proposed, such as protein aggregation, endoplasmic reticulum stress, oxidative stress, mitochondrial impairment, neuro-inflammation, apoptotic cell death, glutamate excitotoxicity, abnormalities in RNA mechanisms, and abnormal function of ubiquitin–proteasome system (UPS) [6].

ALS is typically an adult-onset disease although juvenile forms are present. There are sex-dependent differences in disease development with a slight male predominance [7,8]. ALS can occur in people from all over the world from all ranks of people. Geographical variations have been reported by different population-based studies for the incidence of ALS which ranges 0.6 to 11 cases per 100.000 per year. The prevalence of ALS is between 4.1 and 8.4 per 100.000 persons (reviewed in [9]).

Clinical diagnosis of ALS is difficult in the early period because the patients may not show any upper or lower motor neuron signs [10]. In addition ALS symptoms can be quite heterogeneous and show resemblance to many neurological diseases. Currently the diagnosis is made according to El Escorial Criteria of the World Federation of Neurology and based on complete neurological examination, radiological and electrophysiological investigations [11]. All of these tests may take 3–6 months and cause delay between emergence of early symptoms and diagnosis. It will be possible to prolong the patient’s survival and improve the quality of life with more effective and earlier diagnosis of ALS.

Blood tests are less time consuming and low cost methods compared to other methods for the diagnosis. In addition, the relationship between the values obtained with these analyzes and other variables are very important. This study aims to develop a statistical machine learning model for the prediction of risk of ALS using Parkin protein concentration in blood plasma. For this purpose data was obtained from an experimental study investigating the potential use of Parkin protein as biomarker for the diagnosis of ALS. Patient’s records including age, gender, disease onset, chronic disease information were also obtained from the same study. In this paper, (1) we developed a predictive model using Bayesian networks, (2) examined model performance by comparison with other machine learning methods and (3) created queries based on patient type for evaluation of afore-mentioned variables. In the literature, machine learning methods have been used to examine the genetic architecture of the ALS disease [12]. This study is the first in the literature with its specified features.

## 2. Materials and Methods

**Figure 1:**
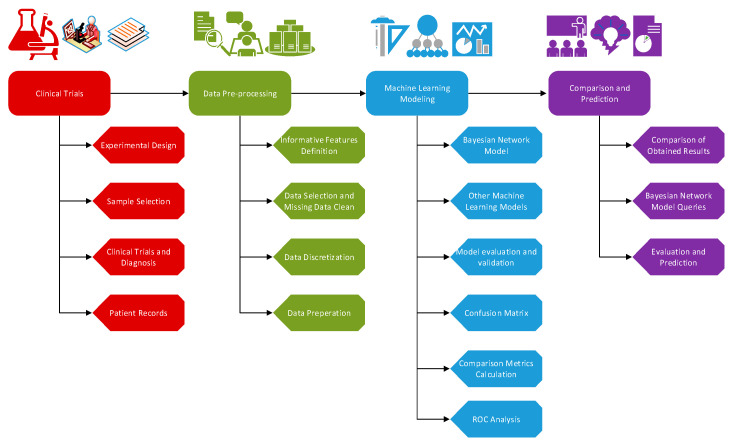
Modeling process with machine learning methods.

In this section, we summarized the data used in the study and explained the basic steps of the experimental design of machine learning methods used to classify and predict the ALS with ALS-related feature interactions. We described the application process of the study in Figure 1.

In Figure 1, first step was clinical trials to obtain experimental data. Second step, after the properties related to ALS were determined, was data pre-processing. In the next step, the data were modeled with Bayesian networks and other machine learning algorithms, and obtained results were compared. Considering the comparison results, last step was evaluation.

### 2.1. Participants

This data set has been obtained from an experimental study investigating the differences on the level of Parkin protein between blood plasma from the ALS patients and other neurological cases including multiple sclerosis, frontal dementia and Parkinson’s disease. There is no missing data in the data set, as the patients amnesia was taken in detail.

The characteristics of the subjects used in the study are given in the Table 1. We confirmed that, sex, age, upper motor neurons (UMN), lower motor neurons (LMN), Bulbar onset types, total number of chronic patience and Parkin level (ng/mL) are related to disease type. Accordingly, 50.5% of the data in the study are from the ALS and 9.3% are from Parkinson’s patients. The Neurological Control (N-Control) group includes people with different neurological diseases other than these diseases. Control group consists of completely healthy individuals. Totally 204 individuals are included. All patients were diagnosed and treated by neurologists at Istanbul Medical University according to El Escorial criteria [11].

### 2.2. Bayesian Networks

Bayesian networks are a graphical modeling approach that models the conditional probabilistic relationships of certain independent variables. In a Bayes network model, nodes correspond to variables, while arrows between nodes show the direct dependency structure between these variables [13]. The direction of the arrow also indicates the direction of the impact.

The probability table for any given *X* node in the network expresses the values given as *X* = *x* for the states of the parents of the node.

(1) $$P(X)=P(X_{1}\ldots X_{n})=\prod_{i=1}^{n}P(X_{i}|Pa(X_{i}))$$

These networks are widely used in medicine and biology [14,15,16,17]. Bayesian networks are very useful in terms of ease of use of posterior probabilities especially in risk assessment studies [17,18]. The ability to refine the network for new information makes the network more useful and adaptive [19]. In addition, it provides to combine the relationships and expert knowledge stated in the literature with the probabilities obtained from the data as a prior probability. In this respect, it is superior to other machine learning methods [20]. Bayesian networks, which are statistically very strong due to the fact that they are based on probability theory. They are accepted as hybrid methods hence they use both classical statistical techniques and heuristic algorithms [21].

### 2.3. Other Machine Learning Methods

Machine learning (ML) methods are a subfield of artificial intelligence (AI) and are becoming increasingly common in clinical research [12,22]. The ML methods are mainly examined in three main categories as semi-supervised, supervised and unsupervised algorithms [23]. Supervised learning methods aim to make predictions about unknown situations (e.g., disease type) based on known situations like age, gender, type of onset [12,23]. Classification, similarity detection and regression are among the most common tasks of supervised machine learning methods [24].

In our study, we examined the following seven popular supervised machine learning techniques with Bayesian Network: Artificial Neural Networks, Logistic Regression, Naïve Bayes Algorithm, J48 Algorithm, Support Vector Machines, KStar Algorithm, and K-Nearest Neighbor Algorithm. We investigate as extensively as possible in terms of computing the best results for each machine learning method.

Artificial Neural Network (ANN), based on its learning and generalization abilities, is one of the learning methods that imitate the human brain. These models basically have a hidden layer and input and output layer. One of the most important advantages is that it works on nonlinear, complex models and missing data. Models are optimized with back propagation algorithms of faults during training. On the other hand, lack of rigid hypotheses found in statistical methods makes the ANN advantageous in modeling [25,26].

Logistic regression (LR) is one of the most widely used methods in biology and health science applications [27]. The LR differs from standard regression models due to the structure of the dependent variable. However, as in linear regression models, the relationships of dependent and independent variables are investigated in the LR. The most important difference here is that the dependent variable in LR is dichotomous. In terms of application, the LR is similar to standard linear regression [28]. In cases where there are more than two situations, the LR can be applied to estimate the dependent variable [29].

Naïve Bayes (NB) Algorithm is one of the most important machine learning methods based on Bayes Rule. This method is a classical Bayesian network based on the independence of variables. Classes to be estimated in the NB method must be independent from each other [30]. This method is one of the supervised learning algorithms. Despite being simple, it produces very successful results in medical applications [31,32].

J48 algorithm is one of the most important decision tree algorithms decision trees include popular machine learning algorithms [33]. This algorithm is a modified version of ID3 [34] and c4.5 algorithms [35,36]. While this algorithm uses c4.5, c5.0, and ID3 algorithms to create the decision tree, criteria such as gini index, information gain or entropy reduction are used for estimation [33,36]. Another important feature of it is that it can make predictions by creating a smaller tree compared to other decision trees. This enables the J48 algorithm to produce more successful results than its counterparts [37].

Support Vector Machines (SVMs) are statistical algorithms that use statistical learning theory to produce a consistent estimator using available data [25]. It tries to divide the data into two basic categories. The n-dimensional hyperplane is produced for this reason [38]. Basically, if linear separation of data is possible, system optimization is done the linear SVM. If not possible, quadratic optimization is provided with the non-linear SVM [38,39,40]. Models use kernel functions for this. The selected kernel function affects the performance of the system. Different results can be obtained with different kernel functions.

KStar algorithm is one of the Instance-based learning algorithms in the WEKA program [41]. It is a method that automatically reveals the number of clusters when the number of clusters is unknown [42]. This algorithm uses entropy as a measure of distance [43]. In this respect, the algorithm is similar to the kNN algorithm that uses entropy as a measure of the distance of the data [44].

The k-Nearest Neighbor Algorithm (k-NN) determines the classification of data according to its closest neighbors. This algorithm is one of the most popular algorithms in data mining work [41]. It is preferred because of simplicity and ease of understandability [45]. The similarity function with the k parameter value in the algorithm affects the performance [46]. It calculates the probability of a data considered to be included in the class of its neighbors based on the status of its nearest neighbor. In this respect, it is superior to NN, which is a completely black box. However, it is difficult to determine the distance between neighbors [25].

### 2.4. Classification Criteria

There are a variety of criteria that can be used to compare the performance of the ML models, the choice of which depends on the structure of the data and nature of the task [12,38,41]. In our study, the numbers of samples in each class are different from each other. In addition, while there are generally two classes in the ML studies, we had four different classes in this study. Increasing the number of classes can affect the results [47]. Since some methods used to evaluate the results are susceptible to unbalanced data, criteria such as Geometric Mean and Youden’s index were also used in the evaluation [48].

The criteria used to determine the algorithms that are effective in this section are given in the Table 2. These criteria were given as Accuracy (ACC), Geometric Mean (GM), Error Rate (ERR), Precision (PREC), Sensitivity (SENS), Specificity (SPEC), F-Measure (FM), Matthew’s correlation coefficient (MCC), Youden’s index (YI), Kappa (κ), False Positive Rate (FPR), and Receiver Operating Characteristic (ROC) Area. Calculation of these formulas is possible by using True positive (TP), True negative (TN), False positive (FP), and False negative (FN) values. Given TP; correct positive prediction, FP; incorrect positive prediction, TN; correct negative prediction, and FN; incorrect negative prediction values are obtained from confusion matrixes.

Accuracy reflects the ratio of true positive and true negative predictions within the total model estimates. The geometric mean is a metric that determines the balance between the results of both the majority and minority subgroups in classification [49]. Accuracy is affected by the changes in the class distribution, but geometric mean is not. For this reason geometric mean is more suitable for the imbalanced dataset [48]. The error rate is complementary to the accuracy. Unlike the measure of accuracy, this metric shows the number of misclassified samples for both positive and negative classes. Precision represents how many positive predictions were genuinely positive for the model. Sensitivity and specificity, representing true positive and true negative rates, are complementary to each other. Sensitivity, also known as the true positive rate, is the ratio of the number of correct positive samples to the number classified as positive, while specificity is the ratio calculated in the same way for negative samples [50].

The equilibrium between precision and sensitivity is represented by the F-Measure. Higher F-Measure indicates good classifier performance. This value is also equal to the harmonic mean of sensitivity and precision [51]. The Matthew’s correlation coefficient is the comparison coefficient that is least affected by unbalanced data and calculates the correlation between observed and predicted classifications. Youden’s index assesses the misclassifications potential of a classifier. The accuracy that can be obtained entirely by chance is calculated by Kappa [52].

The Receiver Operating Curve plots the sensitivity against 1-Specificity to determine an appropriate balance between true and false positive rates. ROC curve is one of the important comparison criteria in clinical studies. This method uses the area under the curve drawn in comparing the subclasses. The larger sum of the AUC shows better classification results [53].

Also, 5-fold cross validation has been preferred for generating estimation results in analyzes. The available data was divided into five, the first four pieces were used for educational purposes and the last piece was used for testing [51]. 5-fold cross-validation is one of the commonly used validation methods to increase model robustness [22].

## 3. Results

### 3.1. Bayesian Network Model

**Figure 2:**
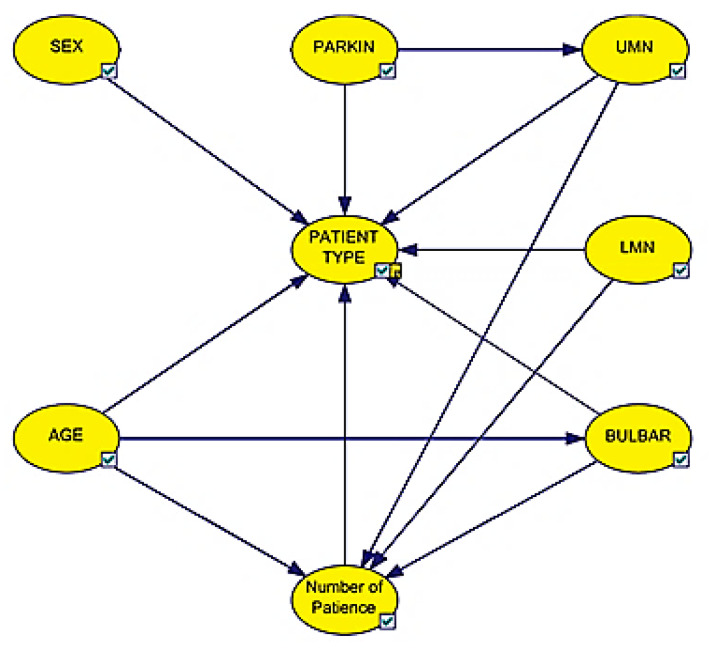
Bayesian network model of dataset.

The Bayesian network model obtained from the data used is given in Figure 2. Arrows show the relationship between variables in the network. The direction of the arrow also indicates the direction of the impact. The network was created using GeNIe 2.1 Academic version. GeNIe is a machine learning program based on Bayesian networks [54].

According to the Bayesian network model, the types of involvement, age, gender, Parkin protein density and the number of diseases directly affect the type of disease. In addition, it is observed that the types of involvement affect the number of diseases. Since there is at least one disease in people except the control group, it is expected that the involvement will affect the number of diseases. It is known that one of the most important symptoms in the ALS disease is UMN involvement. In the model we obtained as a result of the analysis, it was observed that the Parkin protein density affects the UMN involvement.

### 3.2. Comparison Results of Methods

Other machine learning programs that were utilized for comparison were obtained with the WEKA program. This program is Java-based open source software, created by the University of Waikato to facilitate the realization of the ML algorithms [41].

Classification performances of the algorithms according to the classification criteria stated previously are given in Table 3 and Table 4. The generalized results are shown in Table 3 and the results obtained for each class are shown in Table 4. The best classification results according to the criteria are marked in bold.

When the results are examined in general, it has been seen that Bayesian network produces more successful results than other methods. It has been revealed that the Bayes network classifications with little differences. On the other hand, it has been observed that the results of other machine learning methods were close to each other. Polykernel is used for the SVM. For the k-NN, it was seen that the most successful result was obtained with the closest 1 neighbor.

When Table 3 is examined, it is seen that the ACC of Bayesian network is 88.7%. It is observed that the success rates of other methods are approximately 80%. Since Sensitivity and Precision values are the same in the general comparison table, precision values are not included in the table. Specificity value, which expresses confidence in results, shows correctly positively classified variables [55] and this ratio gave high values in all methods. However, the lowest false positive classification rate (0.024) was obtained with Bayesian networks. The same results are also valid for the weighted ROC value.

**Figure 3:**
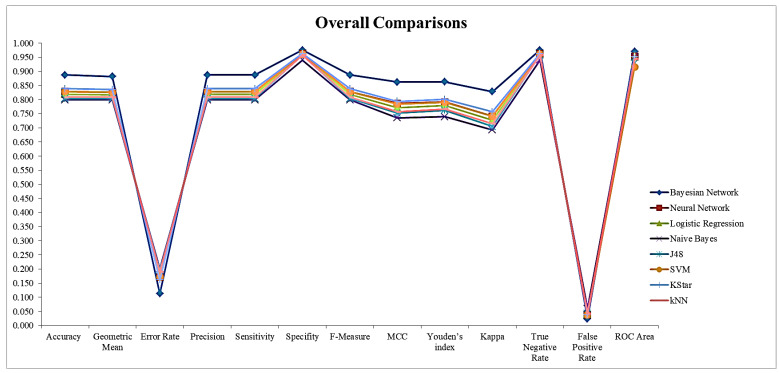
Overall comparison of methods.

Graphical comparison of the results is given in Figure 3. When the graph is examined, it is observed that the compared machine learning methods are close to each other and that Bayesian network produces better results than the compared machine learning algorithms.

Comparison should be made for subclasses as well as general comparison of methods. The results of comparison obtained for each subclass are given in Table 4. Accordingly, Bayes network produced more successful results in the ALS estimation than other methods. It was observed that all individuals in the ALS patient group were classified correctly. The results obtained with the SVM and the NN are also close to these values. It can be proposed that all methods yield successful results in predicting the ALS patients. In addition, it is very important to estimate the individuals in other classes.

When the results for the control group were examined, it has been seen that Bayesian network gives the highest ACC value with 0.917. On the other hand, J48 algorithm produced the best results according to GM (0.902), SENS (0.929), and YI (0.805) criteria. However, Bayesian network showed the best fit (0.747) with Kappa value [56] between data and forecast results. In addition, the best results for other criteria for the control group were produced by the Bayesian network.

Bayesian network has produced more successful results than other methods according to all comparison criteria for the Neurological Control group, as in the ALS group. For this group, the Bayesian Network’s ACC value has been found as (0.902). The Kappa values of other methods indicate that the results obtained are random, while the Kappa value (0.677) was found for Bayesian network. 

Similar results to the control group were obtained for the last group, Parkinson. The Kstar algorithm produced the best results according to the GM (0.920), SENS (0.895) and YI (0.841) criteria. However, it has been seen that the results obtained for Bayesian network are close to these values.

**Figure 4:**
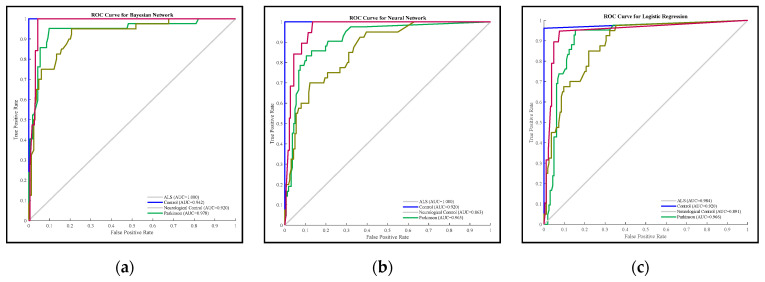

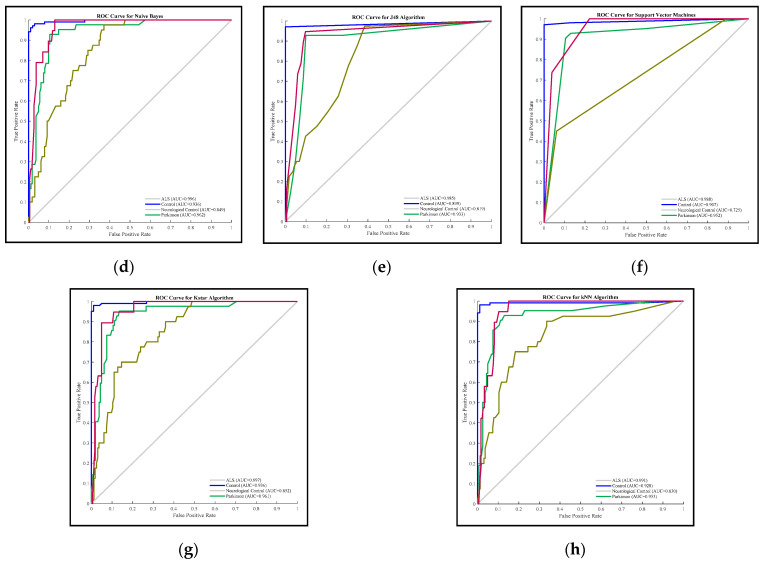
ROC Analysis Results of Methods; (**a**) Bayesian Network, (**b**) Neural Network, (**c**) Logistic Regression, (**d**) Naive Bayes, (**e**) J48, (**f**) SVM, (**g**) KStar, (**h**) kNN.

The ROC curves and the AUC values of the methods are given in Figure 4. According to these values, the AUC value of Bayesian network for each class is higher than other methods. This result supports the values given in Table 4.

### 3.3. Queries of Bayesian Network Model

One of the most important features of Bayesian networks is that predictions can be made by creating queries with the information and data available [20]. While the known variables are included as evidence, the predicted variables are taken as target nodes. When a new person in one of the disease groups is considered, the questions about the status of other variables are given in Table 5.

When the probability values given in Table 5 are examined, in the absence of any prior knowledge, probability values of the persons are P(*Patient Type = ALS*) = 0.340; P(*Patient Type = Control*) = 0.252; P(*Patient Type = Neurological Control*) = 0.257 and P(*Patient Type = Parkinson*) = 0.151. From these given values, the conditional probability value obtained for gender is shown in Equation (2).

(2) $$\begin{array}{l} P\;\left(SEX\;=\;Female\;|\;Patient\;Type\;=\;ALS\right)=\;0.382\\ P\;\left(SEX\;=\;Male\;|\;Patient\;Type\;=\;ALS\right)\;=\;0.618 \end{array}$$

According to this result, it is understood that the ALS disease is seen 62% in men and 38% in women. In addition, the ALS disease is expressed largely as an adult-onset disease in the literature [9]. In this part, it was found that 88.1% of the ALS patients were older than 36 years. Furthermore, it was predicted that 54.8% of the ALS patients and 60.5% of Parkinson’s patients were older than 52 years.

(3) $$\begin{array}{l} P\;\left(UMN\;=\;No\;|\;Patient\;Type\;=\;ALS\right)=\;0.273\\ P\;\left(UMN\;=\;Yes|\;Patient\;Type\;=\;ALS\right)\;=\;0.727 \end{array}$$

when the information given in Equation (3) is examined, it is predicted that 72.7% of the ALS patients have the UMN type onset involvement. In addition, it is understood that 82.2% of the patients do not have the LMN and 85.3% have no bulbar onset involvement. However, it was calculated that there were 3.8% of the ALS patients with no involvement. In summary, the probability of having at least 1 type of onset involvement in the ALS patients was predicted 96.2%.

In Table 5, 25.7% of the ALS patients have at least 1 disease other than their own disease. This probability was 19.4% in Parkinson’s patients. This probability was found to be 11.6% in the control group and 11.7% in the neurological control group. Accordingly, it can be thought that different neurological-chronic diseases are related to neurological diseases such as Parkinson’s or ALS.

**Figure 5:**
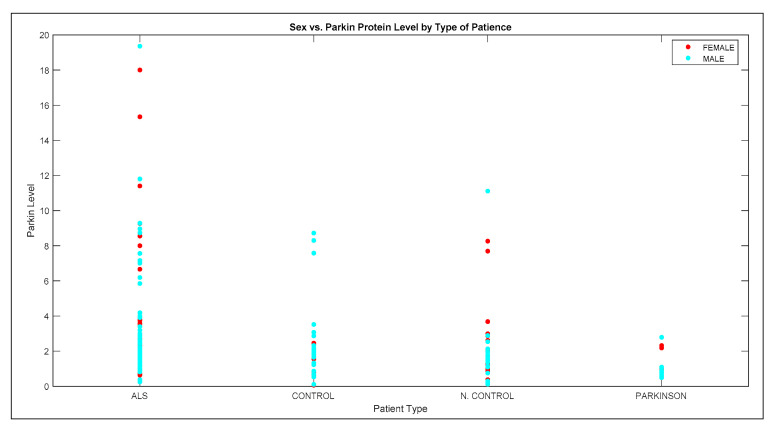
Sex vs. Parkin level (ng/mL) by the type of disease.

Moreover, according to the Parkin level, it is predicted that 75.3% of the ALS patients to be higher than 1.36 (ng/mL). This value is quite different in Parkinson’s patients. When Table 5 is examined, 56.3% of Parkinson’s patients’ Parkin level is lower than 1.36 (ng/mL). Also, Parkin level distribution is given in Figure 5. The protein level differences of the groups are also shown in the graph. Protein level is highest in the ALS patients, but this level is lowest in Parkinson’s patients.

## 4. Discussion and Conclusions

The use of machine learning methods with personal medical records in medical decision-making processes is increasing. In this study Bayesian network—one of the most beneficial ML method in clinical decision-making—has been used for the prediction of ALS, based on differences in the level of a plasma protein, onset, age, sex, and total number of patience. Then results were compared with some popular ML algorithms. To the best of our knowledge, this is the first performance comparison study for Bayesian network model and the ML models for predicting ALS disease using these variables.

Bayesian Networks are one of the probabilistic expert systems that use probability as a measure of uncertainty in order to obtain a graphical structure that best represents the data [57,58]. Since BN uses all the variables in the model, it is easily used in cases where there is missing data [13,59]. With diagnostic reasoning in BN, it is ensured to make a judgement about the patient and the disease by observing various symptoms [60]. Unlike various rule-based ML methods such as NN, LR, SVM, and BN is a method of inference and reasoning. These features allow making queries that reveal cause-effect relationships between variables in the model [13]. The posterior probability values of the network are updated with every new information acquired in BNs. Therefore, the use of BN in prediction problems produces more effective results [61]. The transparency of all relationships in the network structure makes BN advantageous to other ML methods such as k-nn, NN and LR. In addition, it can produce successful results in cases where the data set is small and the number of variables is high [62]. Discretization is main drawback of the BNs which causes loss of information [63]. However, working with discrete data increases the power of accurate prediction regarding classes [64]. All these features have made BN a preferred method in clinical studies [59,62,65,66,67,68].

In this study, unlike the literature, there are three control groups; Parkinson’s disease, neurological control and healthy individuals in the control group. In this way, a comparative result with different control groups containing a large number of subjects improves the applicability of the study in practice.

According to the results of this study, ALS disease is more likely to be seen in men than in women. Various studies have also indicated that gender is an independent variable affecting ALS along with other demographic factors [5,69,70]. Gender was an influential variable and it was confirmed that the ALS disease is more common in males [7,8,71,72,73]. There are studies showing that there is a difference in onset of the disease in ALS patients with different mutations depending on sex [70,74]. Although it is known in which gene some of ALS patients carry a mutation in this study, it has not been taken into consideration. In the future, a similar analysis can be applied to a more homogeneous ALS patient group in terms of mutation.

It has been determined that with the algorithm used in this study, the probability of having ALS will be higher with increasing age. This finding is also consistent with the results of previous studies [75,76]. UMN, LMN and Bulbar are the onset types seen in ALS disease. The probability of having at least one of each kind of onset involvement in the ALS patients was found to be 96.2%. UMN has been determined to be the most common type of involvement. LMN and Bulbar are less common. ALS patients can present together with each a LMN or UMN prevalent phenotype [77]. Previously particular clinical and demographic characteristics of ALS phenotypes have been demonstrated in a population based study with a large epidemiological setting The likelihood of a specific phenotype occurring in different age and gender groups changes. Bulbar phenotype occurs mostly in elderly patients with almost equal incidence rates in the two genders [76].

In particular, the ALS is considered as a multifactorial disease which influenced by environmental and genetic factors. Other neurological diseases that people have can have a small effect on the ALS. It is thought that brain damages and mutations [77] caused by other diseases such as schizophrenia [78], Alzheimer’s disease, Parkinson’s disease, or frontotemporal dementia [79,80] should be associated with the ALS. In our study, the probability of having multiple diseases with the ALS was higher than the control and neurological control groups. Similar results were seen in Parkinson’s patients. Therefore, it will be beneficial to treat patients considering multiple disease situations.

According to all these results, the algorithms we use and the Bayesian network can predict the correct classes with high accuracy rates when information such as the type of involvement of individuals, Parkin protein level, age, and the number of various chronic diseases are considered. Although other machine learning algorithms also produce results with high success, the most important advantage of Bayesian network in this regard is that it can be updated with new additional information and this aspect increases its success. In this respect, it provides more useful results than other machine learning methods such as artificial neural networks showing black box feature in prospective studies, the change of the results should be examined by increasing number of samples and using more variables. The results obtained from statistical and computational methods may be more useful in combination with neuroimaging methods. There is such a study in the literature [81]. A similar approach can be used to classify images of different brain networks as alternative or additional views and the entire MV framework can be further extended to combine imaging with non-imaging views, such as clinical, behavioral, or even genetic multidimensional data, when available from the same subjects.

## Figures and Tables

**Table 1:** Characteristics of patients.

Feature Name	Feature Value	Freq.	%Value
SEX	Female	79	38.7
	Male	125	61.3
AGE	Below 36	29	14.2
	Between 36–52	70	34.3
	Between 52–67	79	38.7
	Upper 67	26	12.7
UMN	No	129	63.2
	Yes	75	36.8
LMN	No	178	87.3
	Yes	26	12.7
BULBAR	No	182	89.2
	Yes	22	10.8
Total Number of Chronic Patience	Five	1	0.5
	Four	1	0.5
	Three	12	5.9
	Two	20	9.8
	One	112	54.9
	None	58	28.4
PARKIN Level (ng/mL)	Upper than 3.74	31	15.2
	Between 2.79–3.74	17	8.3
	Between 2.06–2.79	36	17.6
	Between 1.36–2.06	52	25.5
	Lower than 1.36	68	33.3
Patient type	ALS	103	50.5
	Control	42	20.6
	N-Control	40	19.6
	Parkinson	19	9.3

**Table 2:** Evaluation criteria formulas.

Criteria	Formula
Accuracy	ACC = (TP + TN)/(P + N)
Geometric Mean	GM = sqrt ((TP/(TP + FN)) × (TN/(TN + FP)))
Error Rate	EER = (FP + FN)/(TP + TN + FP + FN)
Precision	PREC = TP/(TP + FP)
Sensitivity	SENS = TP/(TP + FN)
Specificity	SPEC = TN/(FP + TN)
F-Measure	F-Measure = 2 × TP/(2 × TP + FP + FN)
Matthews Correlation Coefficient	MCC = TP × TN − FP×FN/sqrt((TP + FP) × (TP + FN) × (TN + FP) × (TN + FN))
Youden’s index	YI = TPR + TNR − 1
Kappa	Kappa = 2 × (TP × TN – FN × FP) / (TP × FN + TP × FP + 2 × TP × TN + FN^2^ + FN × TN + FP^2^ + FP × TN)
Overall Kappa	Kappa = (p_0_ − p_e_)/(1 − p_e_)
	p_0_ = observed accuracy; p_e_ = expected accuracy
False Positive Rate	FPR = FP/(FP + TN)

**Table 3:** Overall comparisons for methods.

	ACC	GM	ERR	SENS	SPEC	F-M	MCC	YI	Kappa	FPR	ROC
Bayesian Network	0.887	0.882	0.113	0.887	0.976	0.887	0.862	0.863	0.828	0.024	0.970
Neural Network	0.828	0.826	0.172	0.828	0.963	0.828	0.787	0.791	0.741	0.037	0.953
Logistic Regression	0.819	0.817	0.181	0.819	0.960	0.819	0.772	0.778	0.727	0.040	0.951
Naive Bayes	0.799	0.800	0.201	0.799	0.940	0.799	0.736	0.739	0.693	0.060	0.951
J48	0.804	0.804	0.196	0.804	0.958	0.804	0.752	0.762	0.705	0.042	0.930
Support Vector Machine (SVM)	0.828	0.826	0.172	0.828	0.962	0.828	0.784	0.790	0.741	0.038	0.916
KStar	0.838	0.835	0.162	0.838	0.963	0.838	0.794	0.801	0.756	0.037	0.952
k-Nearest Neighbor (k-NN)	0.809	0.808	0.191	0.809	0.958	0.809	0.756	0.766	0.715	0.042	0.943

**Table 4:** Comparisons of Methods for ALS, Control, Neurological Control and Parkinson Disease.

		ACC	GM	ERR	PREC	SENS	SPEC	F-M	MCC	YI	Kappa
ALS	Bayesian Network	1.000	1.000	0.000	1.000	1.000	1.000	1.000	1.000	1.000	1.000
	Neural Network	0.985	0.985	0.015	1.000	0.971	1.000	0.985	0.971	0.971	0.971
	Logistic Regression	0.975	0.975	0.025	1.000	0.951	1.000	0.975	0.952	0.951	0.951
	Naive Bayes	0.971	0.970	0.029	0.962	0.981	0.960	0.971	0.941	0.941	0.941
	J48	0.980	0.980	0.020	1.000	0.961	1.000	0.980	0.962	0.961	0.961
	SVM	0.980	0.980	0.020	1.000	0.961	1.000	0.980	0.962	0.961	0.961
	Kstar	0.975	0.976	0.025	0.990	0.961	0.990	0.975	0.951	0.951	0.951
	k-NN	0.956	0.956	0.044	0.990	0.922	0.990	0.955	0.914	0.912	0.912
Control	Bayesian Network	0.917	0.874	0.083	0.791	0.810	0.944	0.800	0.747	0.754	0.747
	Neural Network	0.882	0.813	0.118	0.714	0.714	0.926	0.714	0.640	0.640	0.640
	Logistic Regression	0.868	0.845	0.132	0.642	0.810	0.883	0.716	0.638	0.692	0.631
	Naive Bayes	0.882	0.854	0.118	0.680	0.810	0.901	0.739	0.668	0.711	0.664
	J48	0.887	0.902	0.113	0.661	0.929	0.877	0.772	0.718	0.805	0.700
	SVM	0.892	0.897	0.108	0.679	0.905	0.889	0.776	0.719	0.794	0.706
	KStar	0.907	0.888	0.093	0.735	0.857	0.920	0.791	0.735	0.777	0.732
	k-NN	0.912	0.891	0.088	0.750	0.857	0.926	0.800	0.746	0.783	0.744
Neurological Control	Bayesian Network	0.902	0.816	0.098	0.778	0.700	0.951	0.737	0.678	0.651	0.677
	Neural Network	0.848	0.738	0.152	0.615	0.600	0.909	0.608	0.513	0.509	0.513
	Logistic Regression	0.848	0.668	0.152	0.655	0.475	0.939	0.551	0.471	0.414	0.462
	Naive Bayes	0.809	0.568	0.191	0.519	0.350	0.921	0.418	0.317	0.271	0.309
	J48	0.819	0.532	0.181	0.571	0.300	0.945	0.393	0.320	0.245	0.299
	SVM	0.843	0.650	0.157	0.643	0.450	0.939	0.529	0.449	0.389	0.439
	KStar	0.853	0.670	0.147	0.679	0.475	0.945	0.559	0.485	0.420	0.474
	k-NN	0.833	0.661	0.167	0.594	0.475	0.921	0.528	0.432	0.396	0.428
Parkinson	Bayesian Network	0.956	0.903	0.044	0.727	0.842	0.968	0.780	0.759	0.810	0.756
	Neural Network	0.941	0.869	0.059	0.652	0.789	0.957	0.714	0.686	0.746	0.682
	Logistic Regression	0.946	0.898	0.054	0.667	0.842	0.957	0.744	0.721	0.799	0.715
	Naive Bayes	0.936	0.840	0.064	0.636	0.737	0.957	0.683	0.650	0.694	0.648
	J48	0.922	0.832	0.078	0.560	0.737	0.941	0.636	0.600	0.677	0.593
	SVM	0.941	0.842	0.059	0.667	0.737	0.962	0.700	0.669	0.699	0.667
	KStar	0.941	0.920	0.059	0.630	0.895	0.946	0.739	0.721	0.841	0.707
	k-NN	0.917	0.857	0.083	0.536	0.789	0.930	0.638	0.607	0.719	0.593

**Table 5:** Bayesian network model queries for patient type.

Target Node (s)	Target Value	Evidence (Patient Type)			
		ALS	Control	N-Control	Parkinson
None	None	0.340	0.252	0.257	0.151
AGE	Below 36	0.119	0.249	0.106	0.078
	Between 36–52	0.333	0.419	0.298	0.316
	Between 52–67	0.437	0.232	0.449	0.430
	Upper 67	0.111	0.100	0.148	0.175
SEX	Female	0.382	0.342	0.401	0.452
	Male	0.618	0.658	0.599	0.548
PARKIN Level (ng/mL)	Upper than 3.74	0.244	0.107	0.098	0.114
	Between 2.79–3.74	0.078	0.072	0.100	0.084
	Between 2.06–2.79	0.192	0.200	0.159	0.131
	Between 1.36–2.06	0.238	0.279	0.340	0.107
	Lower than 1.36	0.247	0.343	0.303	0.563
UMN	No	0.273	0.840	0.844	0.733
	Yes	0.727	0.160	0.156	0.267
LMN	No	0.822	0.912	0.913	0.852
	Yes	0.178	0.088	0.087	0.148
BULBAR	No	0.853	0.924	0.925	0.872
	Yes	0.147	0.076	0.075	0.128
Total Number of Patience	None	0.040	0.696	0.288	0.075
	One	0.704	0.189	0.595	0.731
	Two	0.140	0.060	0.059	0.101
	Three	0.103	0.042	0.045	0.070
	Four	0.008	0.008	0.007	0.013
	Five	0.006	0.006	0.006	0.010
